# Hospitals and the Insurance Bill

**Published:** 1911-07-08

**Authors:** 


The Hospital

A Journal of

The Medical Sciences and Hospital Administration.

New Series. No. 232, Vol. IX. [No. 1304, Vol. L.] SATURDAY, JULY 8, 1911

### HOSPITALS AND THE INSURANCE BILL.

A committee including representatives of the British Hospitals Association and the Central Hospital Council for London have prepared a memorial to the Chancellor of the Exchequer which sets forth the claims of the voluntary hospitals, the injury likely to accrue to them through the passing of the Bill, and the demand that the benefits to be conferred by the Bill should be extended to cover treatment in general or special hospitals, and that such hospitals should be recouped the expenses which they may actually incur by the treatment of persons voluntarily or compulsorily insured by the Bill. The voluntary hospitals are the backbone and mainstay of the poor and humbler members of the population in these islands when seriously ill or suffering from accidents. They at present meet the demands under this head at an annual cost of some four million pounds, and that sum does not include all the charges, or any sum for interest on the original cost of the hospital buildings. The voluntary hospitals guarantee the efficiency of all the medical schools, which supply the only opportunities we possess for training to the physicians and surgeons of the country. We are dependent upon the voluntary hospitals for development in research and the ever-widening possibilities of medical and surgical treatment. It is therefore of paramount importance, nay, an absolute necessity, to the well-being of the overwhelming majority of the population of these islands, that the voluntary hospitals shall be protected from injury and loss through the passing of the National Insurance Bill.

The voluntary hospitals were established to provide medical and surgical treatment and care, with adequate nursing, for those members of the population especially who can maintain themselves and their families when in good health, but who cannot meet the charges incidental to illness or accident. No doubt a large number of people at present receive relief from these institutions who have no real claim for free medical relief on the basis of the original constitution of the voluntary hospital. We are therefore to-day in the position to provide, with the co-operation of the Chancellor of the Exchequer, that instead of the National Insurance Bill weakening or injuring the voluntary hospitals it shall be the means of enabling the managers to confine their free relief to those persons alone who are entitled to receive it, whilst all patients insured by the Bill who may need hospital care will henceforward be paid for *pro rata* by the Government. Here is the simple solution of all the difficulties with which the voluntary hospitals are threatened by the National Insurance Bill. Here too is the solution of a host of difficulties which must otherwise beset national insurance when established by Act of Parliament, for it will enable all insured persons to obtain all the hospital care and nursing which they need, while the voluntary system of hospital support is left untouched. All voluntary gifts to hospitals would in such circumstances be devoted entirely to the relief of those members of the community entitled to receive it, because they come within the definition just given of the people who are by right entitled to free medical relief in a voluntary hospital.

It would be fatal to the voluntary principle for the Government to contribute a certain arbitrary sum to each hospital each year, but where the State requires the provision of hospital care for an insured person under the new Act, there the State must clearly undertake to bear the actual cost of treating all such cases in a voluntary hospital. Sir William Cobbett, who is a very able and loyal worker for voluntary hospitals, is reported to have said that the hospitals do not ask for State aid, but that they may have a share of the worker's fourpence and the employer's threepence. We can therefore claim Sir William Cobbett as supporting the plan we have just laid down, but we think he was a little unfortunate in this suggestion, for were it to be acted upon or even to be advocated by the hospitals, it must bring the managers into conflict with many interests, when any such disaster can be readily obviated. It is no part of the business of the voluntary hospitals to tell the Government from what source it shall provide the *pro rata* payment to meet the actual cost of hospital treatment for insured persons. That is a matter which it is the duty of the Government to arrange. All that hospital managers can reasonably require or are entitled to is the *pro rata* payment for every patient committed to their care by the State. We take it that, should the plan here suggested be followed, the *pro rata* payment must include some sum to defray the cost of medical attendance.

The voluntary principle can be maintained and strengthened, providing clauses 8 and 15 of the Bill are amended so as to provide for the *pro rata* payment to which every voluntary hospital who admits insured persons to treatment is clearly entitled. It is further essential that clause 17 should be deleted or modified by the omission of the authority given to an approved society to subscribe to hospitals and other charitable institutions. It will never do for hospitals to accept relatively small subscriptions from an approved society where they are accompanied, as they would be accompanied, by the declared or implied understanding that all members of such a society are entitled to free medical relief. Such an arrangement is one which the voluntary hospitals cannot afford to enter into, and which on moral grounds is wholly wrong as tending to pauperise the recipients. Mr. Lloyd George has a great opportunity to render a national service by co-operating with the voluntary hospitals to enable them to eliminate all hospital abuse and at the same time to co-operate with the State on a system of *pro rata* payments so as to ensure that at the minimum of cost the maximum of efficient hospital treatment shall be forthcoming everywhere for all insured persons who may need it. Once this principle is admitted, as it must be admitted, by the Government and the hospitals, all the rest is simple. We therefore hope that Mr. Lloyd George will agree to the relatively slight modifications in his Bill which will readily affect these essential changes whereby the whole system of hospital relief can be made to attain the maximum of efficiency, whilst the voluntary system of hospital support will be strengthened and recognised, as it ought to be, in the best interests of all classes.

